# Evaluating Post-Earthquake Building Safety Using Economical MEMS Seismometers

**DOI:** 10.3390/s18051437

**Published:** 2018-05-05

**Authors:** Ting-Yu Hsu, Ren-Cheng Yin, Yih-Min Wu

**Affiliations:** 1 Department of Civil and Construction Engineering, National Taiwan University of Science and Technology, Taipei 10607, Taiwan; 2 Department of Geosciences, National Taiwan University, Taipei 10617, Taiwan; tim0306028@gmail.com (R.-C.Y.); drymwu@ntu.edu.tw (Y.-M.W.)

**Keywords:** post-earthquake, accelerometer, seismometer, P-Alert, building safety

## Abstract

The earthquake early warning (EEW)-research group at National Taiwan University has been developing a microelectromechanical system-based accelerometer called “P-Alert”, designed for issuing EEWs. The main advantage of P-Alert is that it is a relatively economical seismometer. However, because of the expensive nature of commercial hardware for structural health monitoring (SHM) systems, the application of SHM to buildings remains limited. To determine the performance of P-Alert for evaluating post-earthquake building safety, we conducted a series of steel-frame shaking table tests with incremental damage. We used the fragility curves of different damage levels and the interstory drift ratios (calculated by the measured acceleration of each story using double integration and a filter) to gauge the potential damage levels. We concluded that the acceptable detection of damage for an entire building is possible. With improvements to the synchronization of the P-Alert sensors, we also anticipate a damage localization feature for the stories of a building.

## 1. Introduction

During a catastrophic earthquake, hundreds of thousands of buildings could incur damage. When such a massive quantity of buildings sustain sudden damage, at least several months are required in order to dispatch engineers on site to assess the remaining seismic capacity, based mainly on subjective visual examinations, with little support from objective engineering values. Another potential solution is to install a structural health monitoring (SHM) system with sensors on the building, and record the necessary seismic response of the building during an earthquake. By doing so, the building can be seismically evaluated using objective scientific values immediately after an earthquake, without risking the safety of engineers.

Vibration-based damage detection is a promising area in SHM. The dynamic features in the modal domain (e.g., natural frequencies [1], mode shapes and their derivatives [2], and flexibility [3]), in the frequency domain (e.g., frequency response functions [4]), as well as in the time domain (e.g., the residuals of autoregressive models [5]) are typically selected to assess the integrity of the structures. Many researchers have reviewed and discussed the global and vibration-based damage detection methods for structures (e.g., Sohn et al. [6], Carden and Fanning [7], and Brownjohn et al. [8]). The required dynamic features are generally extracted from the measured dynamic response data, and acceleration data are frequently preferred due to the ease of measurement. The dynamic features in the modal domain are probably the most frequently employed for damage detection in the SHM research community because the stiffness reduction can be estimated using these modal parameters.

Many studies have conducted seismic assessments of buildings. Most of the damage indices proposed in the literature are calculated using changes in stiffness. Modal frequencies and mode shapes are frequently identified from the measured acceleration response, after which it is used to estimate the damage indices with a finite element model of the structure (e.g., [9,10]), or only based on the data (e.g., [11,12]). However, the stiffness reduction of a building may not be proportional to the attenuation of building safety. For example, the small initial cracks of reinforced concrete components greatly reduce stiffness, but have little effect on the strength and seismic capacity of the components. Moreover, the modal parameters are based on the assumption of the linear behavior of the building during an earthquake. However, the behavior of a building during an earthquake could be highly nonlinear.

In contrast, fragility curves involve the probability of reaching or exceeding the different states of damage with a peak building response. Because the interstory drift ratio (IDR) has been widely accepted as an indicator for evaluating building damage, the fragility curves of the damage levels and the IDR have been developed [13,14,15], and the U.S. Federal Emergency Management Agency has adopted them in a nationally standardized methodology for earthquake loss assessments [16].

In addition, Naeim et al. [17] applied fragility curves to assess the damage conditions of buildings instrumented with accelerometers under the Strong Motion Instrumentation Program of California. One advantage of using the IDR for damage indices is that it remains valid, even when the building behaves in a highly nonlinear manner. Therefore, the current study assesses post-earthquake building safety using the IDR, but not modal parameters.

Although the obtainment of successful research results have been reported in the SHM literature, due to the expensive nature of commercial SHM hardware, SHM application remains limited. For instance, the initial cost of a typical commercial seismograph with a necessary data acquisition system ranges from approximately $6000 to $12,000 per channel.

Although many wireless sensors have been developed to reduce the cost of an SHM system, because the wireless signals between each wireless sensor are easily blocked by dense walls and floors in a building, the application of wireless sensors is limited chiefly to outside structures (e.g., bridges). Another obstacle stifling the practical application of SHM in structures may be the extent of estimated damage, which does not follow a universal rule. Every individual researcher may define their own damage index for the various approaches applied to different structures. Fortunately, it is possible for engineers to concur to the assessment results pertaining to the extent of damage to building structures if the IDR is estimated.

Over the past 10 years, microelectromechanical systems (MEMS) accelerometers have been tested and applied for recording strong earthquake motion [18]. The earthquake early warning (EEW) research group at National Taiwan University has developed a P-wave alert device named “P-Alert”, which uses MEMS accelerometers to issue EEWs. The cost of P-Alert is approximately $1600 per channel, without necessitating a connection to a data acquisition system. Wu and Lin [19] conducted an 8-week experiment to assess the performance of the P-Alert EEW network located in the Hualien region. Their results encouraged further implementation of the MEMS seismometers for EEW application. Wu et al. [20] proposed a high-density economical sensor network consisting of 400 P-Alert devices deployed for both onsite and regional EEWSs over the entire island of Taiwan. This dense network has facilitated seismological studies throughout Taiwan, particularly for seismic hazard assessment purposes, including those involving regional [21,22] and on-site [23,24] EEW systems, near-real-time shaking maps [25], and permanent displacement induced by earthquakes [26]. The low cost of P-Alert has enabled the rapid implementation of SHM systems for buildings. Because P-Alert was designed not for SHM but for EEWs, the feasibility of these devices for SHM application must be verified. Therefore, the aim of this study is to determine the feasibility of using P-Alert to evaluate post-earthquake building safety.

## 2. Sensor and Method

**Figure 1:**
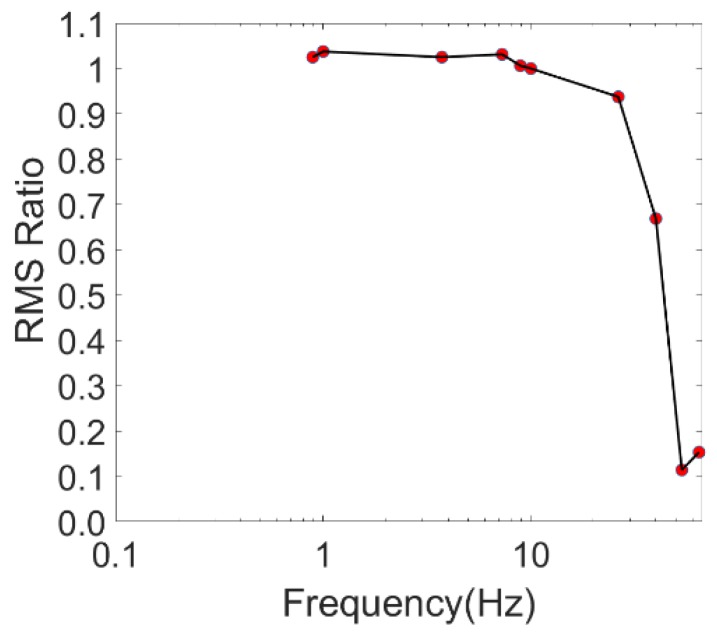
Low-Frequency Accelerometer Calibration System (LFACS) calibration results of a P-Alert sensor.

The P-Alert device is a triaxial MEMS accelerometer. It can record two horizontal and one vertical component of acceleration. Its signal resolution is 16 bits. It has a range of −2 g to +2 g, and a sampling rate of 100 samples per second. P-Alert also has networking capabilities, including the ability to stream real-time acceleration signals to the data collection server, to connect automatically to up to two servers, as well as clock synchronization thanks to the network time protocol (NTP). P-Alert offers 10 Hz and 20 Hz low-pass filters that must select one absolute filter. For EEWs, a 10 Hz low-pass filter is the standard choice. For the SHM cases in this study, we selected a 20 Hz low-pass filter to retain a stronger signal for the building response. The self-noise level of P-Alert is roughly 0.1 gal, which is higher than that of commercial accelerometers. We conducted an instrument calibration experiment using a Low-Frequency Accelerometer Calibration System (LFACS) in the calibration laboratory of the National Center for Research on Earthquake Engineering (NCREE), Taiwan. The certified frequency range of the LFACS in NCREE is 0.8–40 Hz. We compared the measured acceleration results obtained using P-Alert against the results we obtained with the reference accelerometer of the LFACS. The root mean square (RMS) value of the measured Sine waves with different frequencies at 0.8, 1, 2, 5, 8, 10, 15, 20, 30, and 40 Hz was calculated with the same timeframe. We determined the RMS ratio by using the RMS value of P-Alert divided by that of the LFACS. The results revealed that the RMS ratios were close to 1.0, in a range between 0.8 Hz and 10 Hz (Figure 1). The fundamental natural frequency of ordinary buildings lower than 10 stories typically falls within the range of 1–10 Hz. Because most buildings are less than 10 stories high, the quality of P-Alert may meet the requirement for the frequency range of interest, because the chief contributor of the interstory drift is the fundamental natural frequency.

In the present study, we also employed fragility curves to evaluate post-earthquake damage levels by using the IDR as measured by P-Alert. We accepted the definition by the U.S. Federal Emergency Management Agency [16]: five structural damage levels exist (i.e., no damage, slight damage, moderate damage, extensive damage, and complete damage). In this study, the state that is more serious than extensive damage (i.e., complete damage) was merged with extensive damage, because the building is already deemed unsafe. Hence, in our study, we included only four damage states. The conditional probability of falling in or exceeding a particular damage state, DS, with a given IDR, δ, is defined as follows:

(1) $$P(DS|\delta )=\Phi \left[\frac{1}{\beta_{DS}}\ln\left(\frac{\delta }{\delta_{DS}}\right)\right]$$

where $\delta_{DS}$ and $\beta_{DS}$ represent the median and logarithmic standard deviation, respectively, of the IDR at which the building reaches the threshold of the damage state, *DS*; and Φ is the standard normal cumulative distribution function. The median and logarithmic standard deviation of the IDR can be selected from the table provided by the technical manual of earthquake loss assessments [16] for the different building types and seismic design levels.

## 3. Experiment

**Figure 2:**
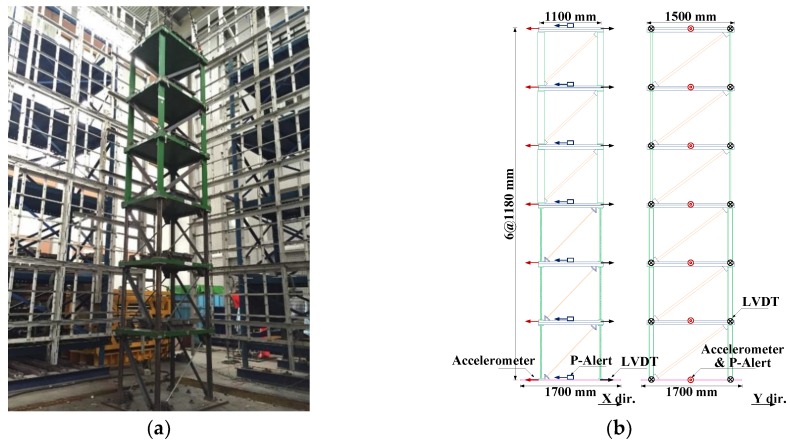
The 1/3-scale six-story steel structure. (**a**) Overview and (**b**) plane views with sensor arrangement.

A 1/3-scale six-story steel structure at the NCREE, Taiwan, was used to experimentally test the sensor and method. As shown in Figure 2, the structure consisted of a single bay comprising a 1.1 m × 1.5 m floor area, with a uniform height of 1.18 m per story. The cross-section of the beams was 50 mm × 100 mm × 6 mm (U-section). For scaled-down specimens of shaking table experiments, the same cross-sections of the elements in each story are typically used. Hence the damage occurs in the lower stories because the forces there are greater. However, due to budget limitations, the upper three stories of the steel building were loaned from another project. Although the story layouts are not typical, the aim to verify the ability to estimate structural safety is still valid without loss of generality. The overall stiffness and strength of these three upper stories were substantially higher compared to their 3 lower counterparts. The cross-section of the columns and bracings of these upper stories measured 150 mm × 25 mm (rectangular section) and 65 mm × 65 mm × 6 mm (L-section), respectively. To confirm that the steel building would sustain damage during the shaking table test, the structure was designed to have less seismic capacity in the lower three stories. The cross-section of the lower columns was 100 mm × 30 mm × 5 mm ×7 mm (H-section). In addition to the L-shaped bracings used in the upper stories, two differently sized tube bracings were used in the *x*-direction in the lower three stories. The outer diameter of these bracings measured 18 mm and 21.7 mm, respectively, with a thickness of 1.2 mm and 2 mm, respectively. For the entire steel building, the beam–floor connections were welded, whereas the beam–column connections and the base–column connections were bolted. The dead load was simulated with lead block units fixed to the steel plate of each floor. The total mass of each floor of the target structure was approximately 962.85 kg, except for the mass of the roof floor, which weighed 903.98 kg. The first natural frequency for the building was approximately 3.5 Hz.

**Figure 3:**
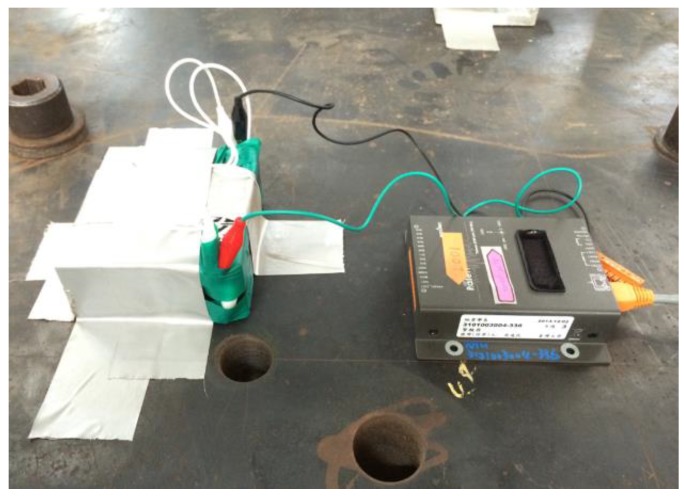
Photograph of installed P-Alert device.

For the wired measurement system of the shaking table facility, Setra141-A accelerometers with an acceleration range of ±4 g and a noise floor of 0.4 mg were used, as were a linear variable differential transformer (LVDT) and the Multiple Pacific Instrument Series PI660-6000 data acquisition chassis. The Setra of the NCREE measurement system was treated as the benchmark for the standard acceleration sensors. Figure 2b shows the location of all the installed sensors, including the P-Alert devices as well as the LVDT and Setra accelerometers. One P-Alert device was fixed to each floor, totaling seven P-Alert devices for each specimen. Figure 3 displays a photograph of an installed P-Alert device on the structure.

Shaking table tests were performed for four specimens with various configurations of bracings and excitations. For all four specimens, the cross-section of all the bracings in the *y*-direction was that of the L-section (labeled “L”), because the excitation of the shaking table occurred only in the *x*-direction, with the El Centro earthquake as input.

**Figure 4:**
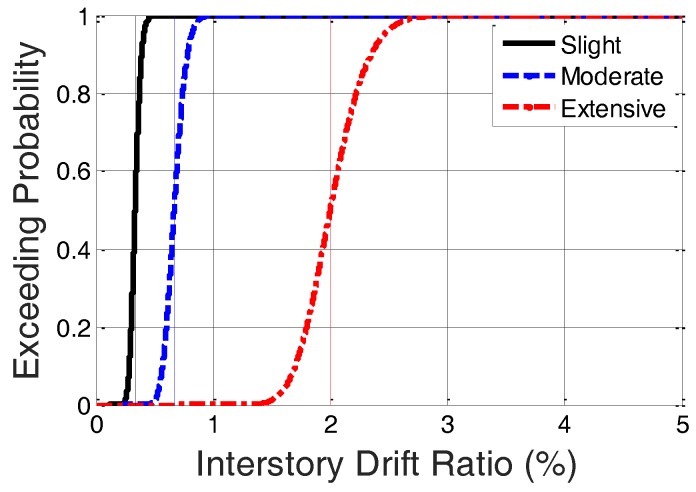
Fragility curve used in this study.

For specimen A, only the first story was installed with a small tube bracing (labeled “T1”), with larger tube bracings (labeled “T2”) installed in the other two stories among the three lower stories. The anticipated maximum peak ground acceleration (PGA) that specimen A could withstand was approximately 400 Gal. Therefore, PGA began at 50 Gal, and was set to increase by 50 Gal with each iteration, until specimen A collapsed. We expected the damage to increase during the sequence of excitations. We also expected the damage to be concentrated in the first story because the seismic capacity of that story was smallest and the story shear was largest. The same sequence of excitations was conducted for model B, but the cross-section of the bracing in the second story was modified to T1. For specimens C and D, the bracing configuration was identical to that of specimen B. The only difference was the program of excitations, where only PGA values equal to 200 Gal, 350 Gal, and 400 Gal were inputted to specimen C, whereas PGA values of 250 Gal, 400 Gal, and 450 Gal were inputted to specimen D in order to investigate the residual seismic capacity after moderate earthquakes. The bracing configuration of the specimens is presented in summary form in Table 1. Based on the category of the specimen that is a 6-story steel-braced frame with a high-code designed seismic level, the median and logarithmic standard deviation of the IDR at which the building reaches the threshold of different damage states can be determined using the table provided by the technical manual of HAZUS-MH. HAZUS-MH provides different values of the median and logarithmic standard deviation of the IDR for different building types. The median values of the IDR used in this study were 0.333%, 0.667%, and 2.0% for slight, moderate, and extensive damage, respectively, whereas the logarithmic standard deviation values of the IDR were 0.124%, 0.124%, and 0.126% for slight, moderate, and extensive damage, respectively. Hence, the fragility curve of the specimen can be drawn as shown in Figure 4.

## 4. Results

Both the Setra and P-Alert accelerometers were much louder at lower frequencies. Moreover, baseline correction was required after integrating the acceleration signals. Therefore, to obtain the displacement time history of each floor, we twice integrated the acceleration time history measured using both Setra and P-Alert before filtering them with a second-order high-pass Butterworth filter.

**Figure 5:**
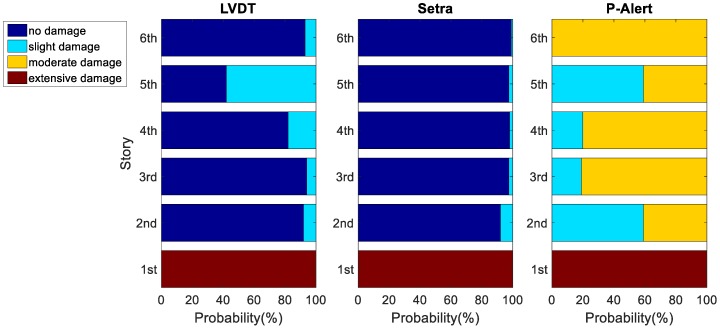
Typical result of Case 3 with peak ground acceleration (PGA) of 400 Gal.

**Figure 6:**
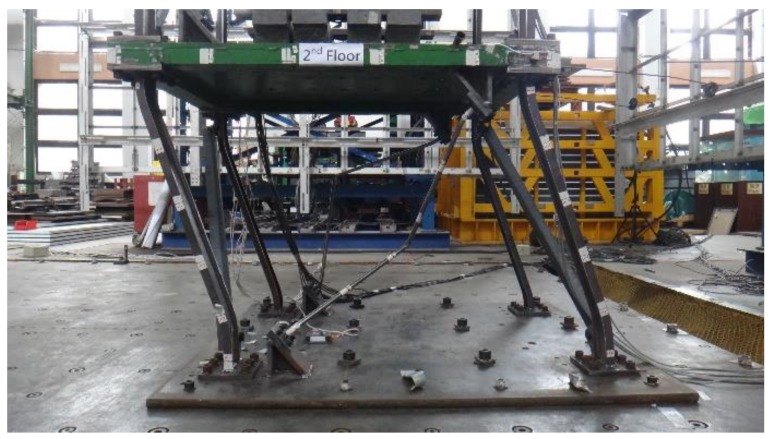
A close-up of Story 1 in Case 3 after the earthquake excitation with a PGA value of 400 Gal.

**Figure 7:**
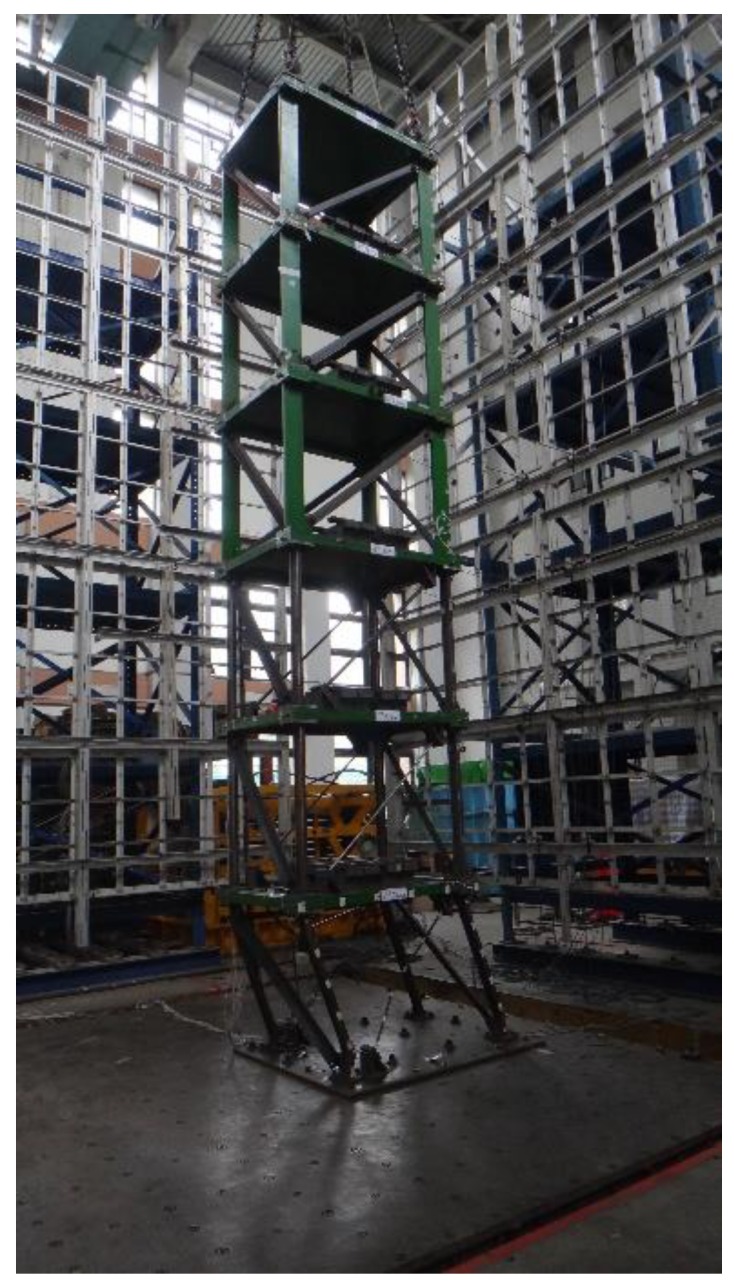
A general view of the entire structure in Case 3 after earthquake excitation with a PGA value of 400 Gal.

Trapani et al. [27] suggested that the cutoff frequency, $f_{c}$, for the filter should be set within the range of 0.2 Hz ≤ $f_{c}$ ≤ 0.5 Hz × f, where f is the fundamental natural frequency of the structure. The cutoff frequency, $f_{c}$, was selected as 0.5 Hz in our study. Afterward, we used the maximum absolute IDR of each story obtained using Setra, P-Alert, and LVDT to calculate the possibility of the damage levels of each story individually. Typical results of Case 3 with a PGA equal to 400 Gal are shown in Figure 5. The *x*-axis represents the probability of structural damage at different levels, and the *y*-axis represents the story. For each story, the damage level with the largest probability was selected to represent the estimated damage level of that story. In this typical result, Stories 1 and 5 were estimated to have sustained extensive damage and slight damage, respectively, and the remaining stories were gauged to have sustained no damage when using the LVDT. The LVDT results are relatively reasonable, because the bracings in Story 1 buckled, and residual displacement was easily noticeable, as is clearly observable in the photograph of Story 1 taken after the earthquake (Figure 6). No obvious signs of damage were noted in the other stories. A photograph of the entire structure after the earthquake is shown in Figure 7.

For all the cases and excitations, the observed extent of damage for every story of the building was relatively consistent with their estimated values obtained using the IDR. When slight damage was estimated using the IDR, a minor buckle for the bracings was usually observed. When moderate damage was expected, the buckle for the bracings and the yielding of the columns were generally observed easily, with a minor residual lateral displacement. When extensive damage was projected, even the residual lateral displacement was evident. The observed maximum damage levels of the building are listed under Table 2. To provide the modal information for the readers of this study, we identified the fundamental natural frequency of each experiment by applying the peak-picking method on the transfer function between the ground and the roof (Table 2).

The LVDT results were treated as the reference. The damage levels estimated using Setra were relatively consistent to that estimated using the LVDT, except that Story 5 was gauged to have sustained no damage when using Setra. We concluded that the damage levels of five of the six stories estimated using Setra were correct because they were identical.

Regarding the damage levels estimated using P-Alert, Story 1 was estimated to have sustained extensive damage, which is identical to the results of both the LVDT and Setra. For Story 5, P-Alert estimated slight damage, which is identical to the LVDT results. For the other stories, the damage levels were overestimated (slight damage and moderate damage). We concluded that the damage levels of only two of six stories estimated using P-Alert were correct. The number of stories correctly gauged using Setra and P-Alert for all the various levels of earthquake excitations in each case are listed in summary form under Table 2. The correct rate of Setra for all the stories in all cases was 85.0%, whereas for P-Alert the correct rate was 49.2%.

However, when we only consider whether the entire structure sustained moderate damage, the damage estimation result still provides useful information for the structure owner. The structure owner can decide to continue operations based on a quick review of certain critical elements after an earthquake provided that the structure did not sustain moderate damage or greater (or request that engineers perform a detailed seismic evaluation if such damage levels are reported). Therefore, we estimated the damage of the entire structure based on the maximum damage of all the stories, and only two levels were considered. The first level of damage was defined as *Safe*, which included no damage and slight damage. The second level of damage was defined as *Unsafe*, which included moderate damage and extensive damage. The results of the correct ratio using the two levels for the entire structure are listed in summary form under Table 3. The correct rate of Setra was 95.0% for the entire structure in all cases, whereas for P-Alert it was 100.0%. This result implies that P-Alert can potentially detect whether an entire structure sustained moderate damage.

Because a high-pass filter filters out residual displacement if displacement is obtained using a double integration setup of the acceleration signals, the maximum IDR could be underestimated using accelerometers, especially under large excitations. Based on this understanding, the correct rate of Setra for damage location in the stories seems reasonable. However, the correct rate of P-Alert for this purpose is relatively low because it does not rely on a centralized data acquisition system. The acceleration signals are digitalized, and the sampling times are recorded simultaneously in each P-Alert sensor. Time synchronization of the P-Alert sensors is based on an NTP that runs every 20 min. The time shift without synchronization every 20 min is approximately 0.13 s, which could cause a large error in the IDR calculation, because it requires perfect synchronization between signals transmitted from two adjacent stories.

**Figure 8:**
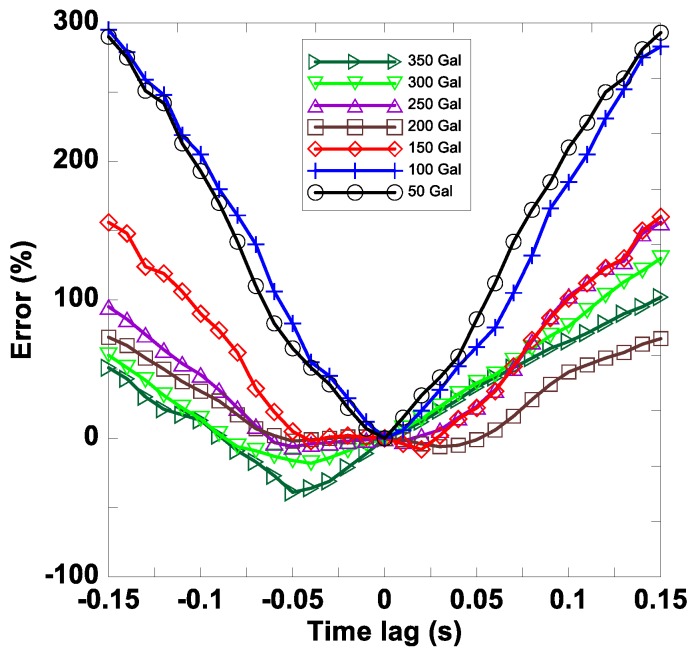
Time difference effects on interstory drift ratio (IDR) estimation.

We examined the effects of the time difference on the IDR at the first story for each excitation in Case 1; the results are shown in Figure 8. The vertical axis represents the error of the IDR, which was calculated using the following equation:

(2) $$\varepsilon_{IDR}{}_{i}=\frac{IDR_{i}-IDR_{0}}{IDR_{0}}$$

where $IDR_{0}$ represents the IDR without a time lag, whereas $IDR_{i}$ represents the IDR with time lag i. The results indicated that the error of the IDR increased rapidly when the time lag increased. Both an overestimation and an underestimation could be obtained when the time lag was small, but overestimation was generally obtained when the time lag was large. The results show that the error caused by the time difference can be reduced substantially if the time difference is small.

**Figure 9:**
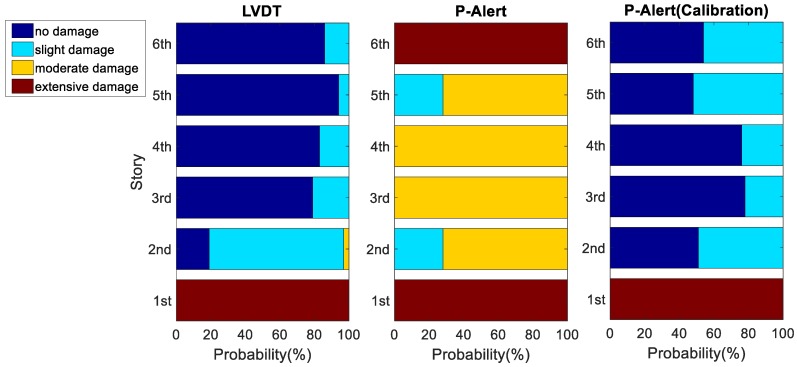
Results of Case 3 with PGA of 350 Gal, obtained with the LVDT, original P-Alert, and pseudosynchronized P-Alert.

Because time synchronization is a critical issue of P-Alert for IDR-reliant post-earthquake safety evaluations, we attempted to synchronize the P-Alert signals offline in order to understand the potential performance of P-Alert under conditions of minimal time difference. A cross-correlation was calculated to estimate the time difference between the P-Alert sensors in different stories, after which it was corrected to create pseudosynchronized acceleration signals. We treated the time lag of the maximum absolute value of the time history of the cross-correlation results as the time difference between the P-Alert sensors in different stories. We assumed that the time difference was a constant between each pair of sensors in the different stories; hence, the corrected IDR was calculated after the time shift of one sensor. One of the worst correct rates of damage level estimation for the stories was demonstrated with a potential pseudosynchronization improvement for P-Alert (i.e., Case 3 with a PGA of 350 Gal, as shown in Figure 9). Figure 9 displays the LVDT results, the original results of P-Alert, and the pseudosynchronized P-Alert results. The figure shows that the pseudosynchronized P-Alert results are much closer to those of the LVDT. The correct rate of the pseudosynchronized P-Alert for all the cases is also listed under Table 2. Despite a number of cases exhibiting little improvement in the correct rate, the overall correct rate increased substantially, to 70.8%. Synchronization was imperfect because we did not know the actual time difference between each P-Alert sensor, and hence, only cross-correlation was used to perform pseudosynchronization. We expect a higher correct rate to be achievable in case of perfect synchronization between the P-Alert sensors.

## 5. Conclusions and Discussion

In this study, we employed the economical P-Alert sensor to evaluate post-earthquake building safety. The fragility curves of different damage levels and the IDR calculated by the measured acceleration of each story using double integration and a filter were used to estimate the potential damage levels. We conducted a series of steel-frame shaking table tests with incremental damage and the IDR to validate the performance of the P-Alert sensors.

Three types of sensors (i.e., LVDT, P-Alert, and Setra) were used to measure the IDR, the results of which were then compared. The IDR results calculated by the LVDT were treated as the reference, and those calculated using Setra were treated as the benchmark of using a standard accelerometer. If the damage levels estimated by P-Alert or Setra were identical to those estimated by the LVDT, the results were considered correct. When the damage level of each story was estimated, although 85.0% of the stories in all the cases were correct using Setra, only 49.2% were correct if P-Alert was used. However, if we limited our aim to detecting whether the entire structure sustained moderate damage, the correct rate for P-Alert was 100.0%. This result implies that, although P-Alert demonstrates a poor indication of damage location, it could anticipate an acceptable level of damage detection for an entire structure.

Based on the results of the calibration tests, a single P-Alert device was verified as reliable for acceleration measurements at a frequency range between 0.8 Hz and 10 Hz. Moreover, in theory, the displacement caused by the lower frequencies is much greater than those resulting from higher frequencies, because the amplitude of the displacement signal is proportional to the inverse of the frequency squared. This implies that P-Alert is suitable for measuring displacement vibration signals contributed by the fundamental natural frequency of most ordinary buildings with fewer than 10 stories, the frequency range of which is between approximately 1 Hz and 5Hz. The low correct rate for the damage level of stories for P-Alert is chiefly due to the time difference between each P-Alert sensor. Based on the substantial improvement to the correct rate, which was observed in the results after the pseudosynchronization of the P-Alert sensors, we recommend improving the synchronization of P-Alert sensors to achieve damage localization for stories.

## Figures and Tables

**Table 1:** Bracing configuration of the four specimens.

Specimen	Story					
	1	2	3	4	5	6
A	T1	T2	T2	L	L	L
B	T1	T1	T2	L	L	L
C	T1	T1	T2	L	L	L
D	T1	T1	T2	L	L	L

**Table 2:** Correct rate for gauging the damage level of each story. (The observed maximum damage level and the identified fundamental natural frequency are also listed.).

Case	Input (gal)	Setra (Correct Stories/Total Stories)	P-Alert (Correct Stories/Total Stories)	Pseudo-Synchronized P-Alert (Correct Stories/Total Stories)	Observed Maximum Damage Level	Identified Fundamental Natural Frequency
Case 1	EL50	6/6	6/6	6/6	None	3.77
Case 1	EL100	6/6	5/6	6/6	Slight	3.62
Case 1	EL150	4/6	3/6	3/6	Moderate	2.88
Case 1	EL200	5/6	3/6	5/6	Moderate	2.78
Case 1	EL250	3/6	0/6	2/6	Moderate	1.82
Case 1	EL300	4/6	2/6	4/6	Extensive	1.11
Case 1	EL350	2/6	4/6	4/6	Extensive	0.75
Case 2	EL50	6/6	6/6	6/6	None	3.42
Case 2	EL100	6/6	6/6	6/6	None	3.41
Case 2	EL150	5/6	5/6	6/6	Moderate	3.21
Case 2	EL200	6/6	1/6	2/6	Moderate	2.73
Case 2	EL250	6/6	1/6	2/6	Moderate	2.05
Case 2	EL300	5/6	3/6	5/6	Extensive	1.15
Case 2	EL350	5/6	3/6	5/6	Extensive	0.81
Case 3	EL200	6/6	1/6	3/6	Moderate	2.45
Case 3	EL350	5/6	1/6	4/6	Extensive	1.16
Case 3	EL400	5/6	2/6	5/6	Extensive	0.82
Case 4	EL250	5/6	2/6	3/6	Moderate	1.95
Case 4	EL400	6/6	2/6	2/6	Extensive	0.85
Case 4	EL450	6/6	3/6	6/6	Extensive	0.76
Total		102/120	59/120	85/120	-	-
Correct rate (%)		85.0	49.2	70.8	-	-

**Table 3:** Correct rate for gauging whether the entire building is safe.

Case No.	Input (gal)	Setra (Estimated/Reference Damage Level)	Correct (Y/N)	P-Alert (Estimated/Reference Damage Level)	Correct (Y/N)
Case 1	EL50	Safe/Safe	Y	Safe/Safe	Y
Case 1	EL100	Safe/Safe	Y	Safe/Safe	Y
Case 1	EL150	Safe/Non-safe	N	Non-safe/Non-safe	Y
Case 1	EL200	Non-safe/Non-safe	Y	Non-safe/Non-safe	Y
Case 1	EL250	Non-safe/Non-safe	Y	Non-safe/Non-safe	Y
Case 1	EL300	Non-safe/Non-safe	Y	Non-safe/Non-safe	Y
Case 1	EL350	Non-safe/Non-safe	Y	Non-safe/Non-safe	Y
Case 2	EL50	Safe/Safe	Y	Safe/Safe	Y
Case 2	EL100	Safe/Safe	Y	Safe/Safe	Y
Case 2	EL150	Safe/Safe	Y	Safe/Safe	Y
Case 2	EL200	Non-safe/Non-safe	Y	Non-safe/Non-safe	Y
Case 2	EL250	Non-safe/Non-safe	Y	Non-safe/Non-safe	Y
Case 2	EL300	Non-safe/Non-safe	Y	Non-safe/Non-safe	Y
Case 2	EL350	Non-safe/Non-safe	Y	Non-safe/Non-safe	Y
Case 3	EL200	Safe/Safe	Y	Safe/Safe	Y
Case 3	EL350	Non-safe/Non-safe	Y	Non-safe/Non-safe	Y
Case 3	EL400	Non-safe/Non-safe	Y	Non-safe/Non-safe	Y
Case 4	EL250	Non-safe/Non-safe	Y	Non-safe/Non-safe	Y
Case 4	EL400	Non-safe/Non-safe	Y	Non-safe/Non-safe	Y
Case 4	EL450	Non-safe/Non-safe	Y	Non-safe/Non-safe	Y
Correct rate (%)		-	95.0	-	100.0
